# PMMA-*g*-OEtOx Graft Copolymers: Influence of Grafting Degree and Side Chain Length on the Conformation in Aqueous Solution

**DOI:** 10.3390/ma11040528

**Published:** 2018-03-30

**Authors:** Irina Muljajew, Christine Weber, Ivo Nischang, Ulrich S. Schubert

**Affiliations:** 1 Laboratory of Organic and Macromolecular Chemistry (IOMC), Friedrich Schiller University Jena, Humboldtstr. 10, 07743 Jena, Germany; irina.muljajew@uni-jena.de (I.M.); ivo.nischang@uni-jena.de (I.N.); 2 Jena Center for Soft Matter (JCSM), Friedrich Schiller University Jena, Philosophenweg 7, 07743 Jena, Germany

**Keywords:** lower critical solution temperature, graft copolymer, thermo-responsive polymer, poly(2-oxazoline), analytical ultracentrifugation, amphiphile, cationic ring-opening polymerization, RAFT polymerization

## Abstract

Depending on the degree of grafting (DG) and the side chain degree of polymerization (DP), graft copolymers may feature properties similar to statistical copolymers or to block copolymers. This issue is approached by studying aqueous solutions of PMMA-*g*-OEtOx graft copolymers comprising a hydrophobic poly(methyl methacrylate) (PMMA) backbone and hydrophilic oligo(2-ethyl-2-oxazoline) (OEtOx) side chains. The graft copolymers were synthesized via reversible addition-fragmentation chain transfer (RAFT) copolymerization of methyl methacrylate (MMA) and OEtOx-methacrylate macromonomers of varying DP. All aqueous solutions of PMMA-*g*-OEtOx (9% ≤ DG ≤ 34%; 5 ≤ side chain DP ≤ 24) revealed lower critical solution temperature behavior. The graft copolymer architecture significantly influenced the aggregation behavior, the conformation in aqueous solution and the coil to globule transition, as verified by means of turbidimetry, dynamic light scattering, nuclear magnetic resonance spectroscopy, and analytical ultracentrifugation. The aggregation behavior of graft copolymers with a side chain DP of 5 was significantly affected by small variations of the DG, occasionally forming mesoglobules above the cloud point temperature (T_cp_), which was around human body temperature. On the other hand, PMMA-*g*-OEtOx with elongated side chains assembled into well-defined structures below the T_cp_ (apparent aggregation number (N_agg_ = 10)) that were able to solubilize Disperse Orange 3. The thermoresponsive behavior of aqueous solutions thus resembled that of micelles comprising a poly(2-ethyl-2-oxazoline) (PEtOx) shell (T_cp_ > 60 °C).

## 1. Introduction

Poly(2-oxazoline)s (POx) can be obtained via a living cationic ring-opening polymerization (CROP). In particular, the hydrophilic poly(2-methyl-2-oxazoline) (PMeOx) and poly(2-ethyl-2-oxazoline) (PEtOx) have recently experienced increasing interest due to their favorable properties for biomedical applications [1]. Similar to poly(ethylene glycol) (PEG), these POx are non-toxic, protein repellent, and exhibit a so-called “stealth effect” in vivo as they are barely recognized by the immune system [2]. This results in elongated blood circulation times and makes them appealing as building blocks for various drug delivery systems. In particular, micellar carriers based on block copolymers have been intensively investigated [3].

The livingness of the CROP enables a straightforward introduction of functional moieties at the POx end groups and, thus, offers a multitude of possibilities to combine POx with other polymer types [4]. In particular, graft copolymers comprising a hydrophobic backbone are interesting for exploration as these polymers represent amphiphiles and could potentially be applied in a similar fashion as block copolymers [5,6]. This concept has been successfully utilized, for e.g., with poly(methyl methacrylate)-*graft*-poly(ethylene glycol) (PMMA-*g*-PEG), to encapsulate anticancer and anti-inflammatory drugs [7,8] or for PEG-grafted polyamides [9]. Graft copolymers comprising hydrophilic POx side chains have been synthesized via grafting from [10,11], grafting onto [12,13], as well as grafting through methods [14,15,16].

As high molar mass PEtOx represents a thermoresponsive polymer, revealing lower critical solution temperature (LCST) behavior in water above 70 °C [17,18], its combination with a hydrophobic graft copolymer backbone can complicate a straightforward micellization because the overall hydrophilicity is decreased. In consequence, the cloud point temperature (T_cp_) of the aqueous solution is lower compared to that of a PEtOx homopolymer. This concept can be exploited in order to tune the T_cp_ [13,14]. On the other hand, the aggregation behavior and conformation below the T_cp_ will be strongly prone to variations of the degree of grafting (DG) and side chain degree of polymerization (DP) [19,20].

**Scheme 1:**
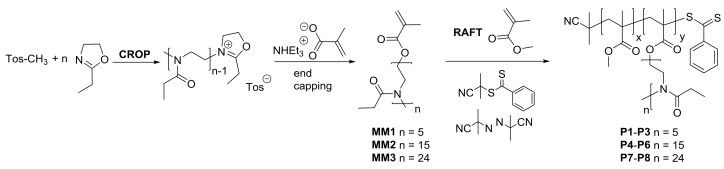
Schematic representation of the synthesis route towards poly(methyl methacrylate)-*graft*-oligo(2-ethyl-2-oxazoline)s (PMMA-*g*-OEtOx). CROP: cationic ring-opening polymerization. RAFT: Reversible addition-fragmentation chain transfer polymerization.

These two factors can be adjusted in a straightforward manner by the copolymerization of PEtOx-based macromonomers containing a methacrylate ω-end functionality. The macromonomer DP can be adjusted by the monomer to initiator ratio during the CROP and will define the side chain DP of the graft copolymers. We selected simple methyl methacrylate as comonomer for the subsequent reversible addition-fragmentation chain transfer (RAFT) copolymerization to form a hydrophobic poly(methyl methacrylate) (PMMA) backbone, adjusting the DG by variation of the comonomer feed ratio (Scheme 1). The resulting PMMA-*g*-OEtOx (OEtOx: oligo(2-ethyl-2-oxazoline)) library was subsequently investigated with respect to LCST and aggregation behavior in aqueous solution by means of turbidimetry, dynamic light scattering (DLS), nuclear magnetic resonance (NMR) spectroscopy, and analytical ultracentrifugation (AUC). Finally, these polymers were used to solubilize the hydrophobic model dye Disperse Orange 3 (DO3).

## 2. Materials and Methods

**Materials.** All chemicals and solvents were purchased from common commercial sources and were used without further purification, unless stated otherwise. 2-Ethyl-2-oxazoline (EtOx) was dried over barium oxide and distilled under argon atmosphere before use. Methyl tosylate (MeTos) was distilled and stored under argon. Azobisisobutyronitrile (AIBN) was recrystallized from methanol. Methyl methacrylate (MMA) was flushed through a short column filled with an inhibitor remover prior to use. For preparative size exclusion chromatography, BioBeads^TM^ SX-1 column material from Bio-Rad was used with tetrahydrofuran (THF) as the eluent. Phosphate-buffered saline was prepared from a powder (Sigma Aldrich, St. Louis, MO, USA) yielding 1 L of 0.01 M aqueous solution (NaCl 0.138 M; KCl 0.0027 M) with a pH value of 7.4 at 25 °C.

**Instrumentation.** The polymerization of EtOx was conducted in a Biotage^®^ Initiator+ microwave synthesizer (Biotage, Uppsala, Sweden). The centrifugation was performed on a ROTINA 380 R (Andreas Hettich GmbH & Co. KG, Tuttlingen, Germany) equipped with a fixed-angle rotor. An Alpha 2-4 LDplus freeze dryer (Martin Christ Gefriertrocknungsanlagen GmbH, Osterode, Germany) was used.

If not noted otherwise, proton nuclear magnetic resonance (^1^H NMR) spectra were recorded at room temperature in CDCl_3_ or D_2_O on a Bruker Avance 300 MHz spectrometer (Bruker, Karlsruhe, Germany) using the residual solvent resonance as an internal standard. The chemical shifts are given in ppm. UV Vis absorption measurements were performed at room temperature using an Analytik Jena SPECORD 250 spectrometer (Analytik Jena AG, Jena, Germany).

Size exclusion chromatography (SEC) was performed on a Shimadzu system (Shimadzu Corp., Kyoto, Japan) equipped with a SCL-10A VP system controller, a SIL-10AD VP auto sampler, a LC-10AD VP pump, a RID-10A refractive index (RI) detector, a CTO-10A VP oven, and a PSS SDVguard/lin S column (5 mm particle size). The system was running with an eluent composed of chloroform/*iso*-propanol/triethylamine [94/2/4] at a flow rate of 1 mL·min^−1^ and at a column oven temperature of 40 °C. The system was calibrated with PMMA standards (410 to 88,000 g·mol^−1^).

Cloud points were determined using a Crystal 16 (Avantium Technologies, Amsterdam, Netherlands) connected to a Julabo FP 40 chiller (Julabo Labortechnik GmbH, Seelbach, Germany) at a wavelength of 500 nm and a heating rate of 1 K·min^−1^.

Sedimentation velocity experiments were performed with a ProteomeLab XL-I analytical ultracentrifuge (Beckman Coulter Instruments, Brea, CA, USA), using double-sector epon or aluminum centerpieces with a 12 mm optical path length. The cells were placed in a An-50 Ti eight-hole rotor. A rotor speed of 42,000 rpm was used. The cells were filled with 420 μL sample solution in water or acetone and with 450 μL water or acetone as the reference. Sedimentation profile scans were recorded at 3 min. intervals with the interference optics (refractive index (RI)) detection system with respect to time. Every fifth scan was used for data evaluation with Sedfit [21]. The experiments were conducted for 24 h at a temperature of T = 20 °C.

Dynamic light scattering (DLS) was performed using a Zetasizer Nano ZS (Malvern Instruments, Herrenberg, Germany). After an equilibration time of 180 s, 3 × 30 runs were carried out at the stated temperature (laser wavelength, λ = 633 nm). The counts were detected at an angle of 173°. The mean particle size was approximated as the effective (Z-Average) diameter obtained by the cumulants method and refers to a spherical solid particle that diffuses at the same speed as the analyte. Each measurement was performed in triplicate.

**General macromonomer synthesis procedure**. The macromonomers (**MM**) were synthesized according to a modified procedure previously published [22]. MeTos, EtOx, and acetonitrile were transferred into a preheated vial under inert conditions. The concentration of EtOx was 4 mol·L^−1^ and the total reaction solution volume was 15 mL. The polymerization was performed in the microwave at 140 °C to reach a ln([M]_0_/[M]_t_) of 4 according to the *k*_p_ value of 0.255 L·mol^−1^·s^−1^. Subsequently, a 1.5-fold excess of methacrylic acid (MAA) and a 2-fold excess of triethyl amine (NEt_3_) were added via syringe through the septum of the vial (excess by reference to the initiator). The reaction solution was kept at 50 °C overnight to allow end functionalization. The reaction mixture was dissolved in chloroform (100 mL), washed with saturated aqueous sodium bicarbonate solution (2 × 100 mL) and brine (2 × 100 mL), dried over sodium sulfate, and concentrated under reduced pressure at 30 °C. The honey-like pale yellow product was stored at −20 °C. A detailed description is provided in the supporting information.

**General synthesis procedure for RAFT copolymerization.** The respective macromonomers **MM1**–**MM3** and MMA were dissolved in ethanol in the desired ratio at an overall monomer concentration [M] of 1 mol·L^−1^. Subsequently, the initiator AIBN and the chain transfer agent 2-cyano-2-propyl benzodithioate (CPDB) were added from adequate stock solutions to achieve a [M]:[CPDB]:[AIBN] ratio of 90:1:0.25, unless noted otherwise. One equivalent of *N*,*N*-dimethylformamide (DMF) with respect to **MM** was added as internal standard. The reaction solution was gently degassed by argon bubbling through the septum of the closed vial for 30 min. An initial sample was taken to determine the monomer conversion by means of ^1^H NMR spectroscopy. The vial was heated to 70 °C in an oil bath overnight and a final sample was taken. The reaction solution was concentrated under reduced pressure and subsequently purified by preparative size exclusion chromatography (BioBeads SX-1 in THF). The desired fractions were concentrated under reduced pressure, the product was precipitated into cold diethyl ether, and dried under reduced pressure at 40 °C. The purified polymers were characterized by means of ^1^H NMR spectroscopy and SEC. A detailed description is provided in the supporting information.

**Solubility and aggregation behavior in water**. For the solubility and aggregation studies in water, the polymers were directly dissolved in the respective solvent at the given concentration and investigated by means of turbidimetry, DLS and AUC (see above).

**Dye encapsulation**. According to the thin film method [23], two separate stock solutions of polymer and dye were prepared in acetone with c(polymer) = 20 mg·mL^−1^ and c(dye) = 1 mg·mL^−1^. The required amounts of both solutions were combined to result in [polymer] to [dye] molar ratios of 1:1, 1:2, 1:3, 1:4, and 1:5. Additional control samples were prepared, containing the same amounts of dye but no polymer. The solvent was evaporated under ambient conditions and further at 40 °C in vacuo overnight. Then, 3 mL of water were added and stirred overnight. The content of each sample was transferred into a centrifuge vial, and the vessel was washed with 1 mL of water. Centrifugation was performed for 1 h at 20 °C with a rotor speed of 11,000 rpm. Afterwards, the supernatant was discarded very carefully with a pipette and freeze dried. The solid material obtained was redissolved in acetone, and the dye uptake was determined by means of UV Vis absorption spectroscopy using a calibration curve generated from a serial dilution of the dye in acetone.

## 3. Results and Discussion

### 3.1. Macromonomer Synthesis

To gain a detailed understanding of the influence of the side chain length on the properties of the graft copolymers PMMA-*g*-OEtOx in solution, a series of macromonomers with different degrees of polymerization (DP) were synthesized by CROP (Table 1). The DP was varied between 5 and 24 by adjustment of the [monomer] to [initiator] ratio (M/I). End capping of the living CROP with triethylammonium methacrylate yielded the OEtOxMA macromonomers **MM1**–**MM3**. Characterization by means of SEC revealed monomodal and narrowly distributed OEtOxMA oligomers with molar masses (M_n_) between 600 and 2500 g·mol^−1^. Characterization by means of ^1^H NMR spectroscopy confirmed the high end group fidelity, making the OEtOxMA suitable macromonomers for RAFT polymerization.

### 3.2. Graft Copolymer Synthesis

**Figure 1:**
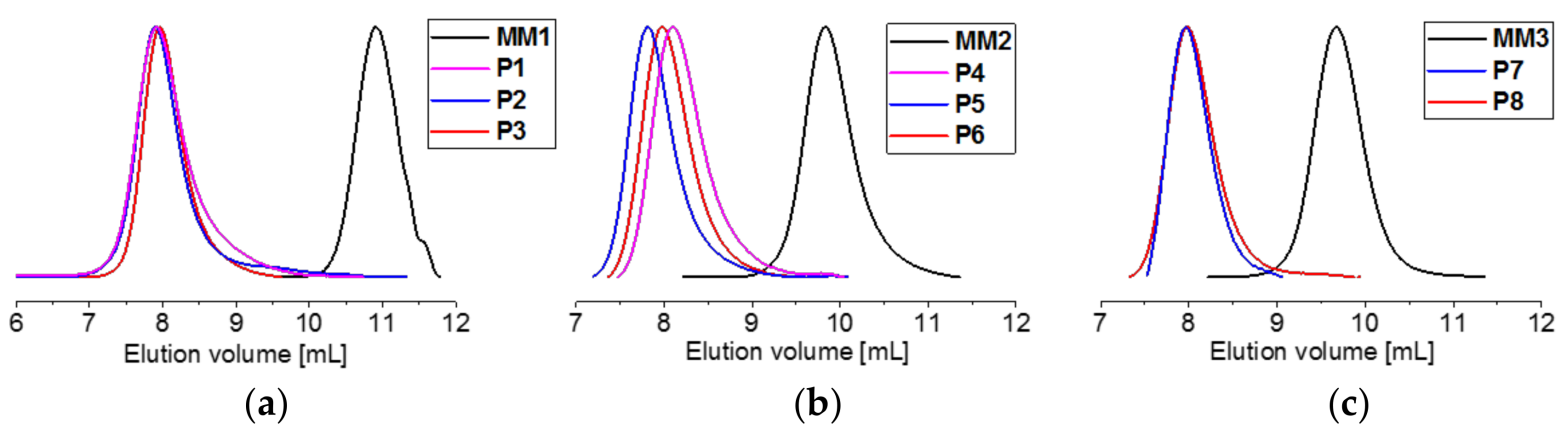
SEC elugrams of PMMA-*g*-OEtOx **P1**–**P3** (**a**), **P4**–**P6** (**b**), **P7**–**P8** (**c**) and the corresponding macromonomers **MM1**–**MM3** utilized within the RAFT polymerization (CHCl_3_, RI detection).

The macromonomer method utilizing **MM1**–**MM3** was applied for the synthesis of the PMMA-*g*-OEtOx **P1**–**P8** via RAFT polymerization (Table 2). MMA served as a hydrophobic comonomer keeping the overall [monomer]/[chain transfer agent] (M/CTA) ratio constant at 90 for **P2**–**P8** to ensure a similar backbone DP. Monomer conversions between 80% and 90% were reached for the majority of the RAFT polymerizations. Since the macromonomers **MM1**–**MM3** vary in molar mass, they also vary in elution volume during the purification by preparative SEC. Due to a larger difference in elution volume between macromonomer and graft copolymer, the purification step was more straightforward for PMMA-*g*-OEtOx comprising shorter side chains, resulting in higher yields. However, the monomodal SEC elugrams confirmed the absence of residual macromonomer in all purified graft copolymers (Figure 1). Except for **P1**, the dispersity (Ð) values of all polymers remained below 1.2. It should be noted that the molar mass values obtained via SEC are relative because an RI detector and PMMA calibration were used (see Section 3.4 for absolute molar masses).

**Figure 2:**
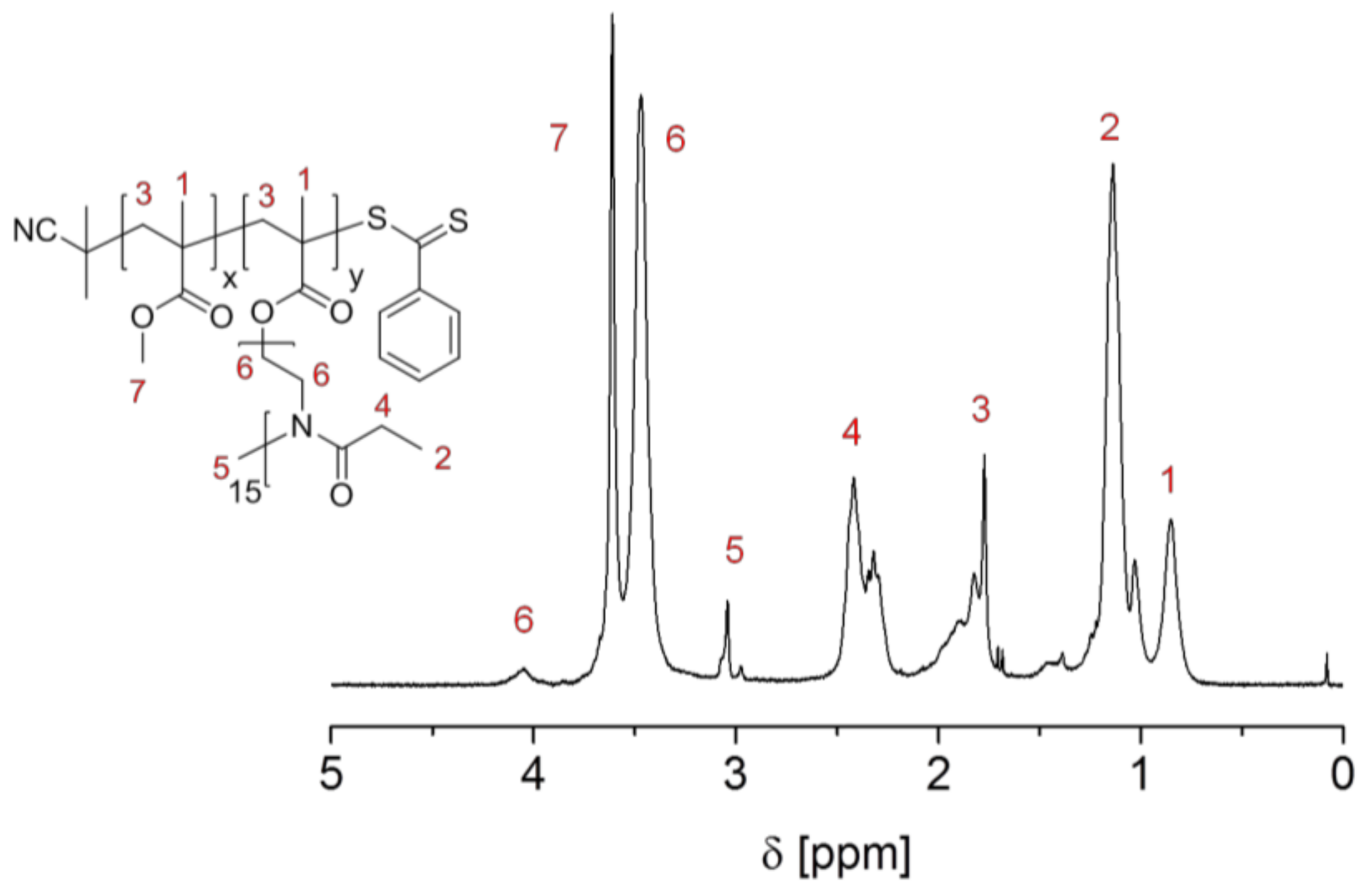
^1^H NMR spectrum (CDCl_3_, 300 MHz) of the PMMA-*g*-OEtOx **P5** and assignment of the peaks to the schematic representation of the structure.

An exemplary ^1^H NMR spectrum of the purified **P5** is shown in Figure 2. All peaks correlate well with the corresponding PMMA-*g*-OEtOx structure. A full spectrum is provided in Figure S1, showing also the presence of the benzodithioate group, which remains detectable despite the high molar mass of the graft copolymer. The composition of the graft copolymers was estimated using the signals “5” corresponding to the side chain methyl end groups and “7” corresponding to the methyl protons of the MMA repeating units in Figure 2. The resulting composition of the graft copolymers, i.e., the DG, differs significantly from the feed ratios of the comonomers. In particular, for the graft copolymers with elongated side chains, **P4**–**P8**, approximately double the amount of MMA was incorporated into the polymer backbone. Apparently, the reactivity of the corresponding macromonomers **MM2** and **MM3** is decreased due to their larger DP resulting in an increased sterical hindrance. To provide a clear picture of the individual macromolecules, the according DP values of the comonomers, i.e., MMA and **MM1**–**MM3** are noted in Table 1.

### 3.3. Thermo-Responsive Behavior

Based on our previous research conducted on PMMA-*g*-EtOx graft copolymers featuring a higher DG and a side chain DP of 5 [14], it was expected that the aqueous solutions of the PMMA-*g*-OEtOx with different side chain length and DG would also exhibit LCST behavior. The temperature dependence of the solubility of the synthesized graft copolymers in water as well as phosphate-buffered saline (PBS) was investigated by turbidimetry measurements. At least two heating-cooling cycles were performed at a heating rate of 1 K·min^−1^ in order to investigate the reversibility of the phase transition. The T_cp_ values were taken at a transmittance of 50%. For a general overview, the cloud point temperatures T_cp_ of **P1**–**P8** were determined in solution at a concentration of 1 and 5 mg·mL^−1^. 

Due to its high content of hydrophobic MMA, **P4** was not soluble in water at 4 °C. All other graft copolymers revealed LCST behavior in aqueous solution (Figures S2–S4). Only a minor heating-cooling hysteresis of the turbidimetry curves of less than 4 °C was observed, which results from the dynamic character of the method [24]. The fact that the cloud point temperatures were constant for all heating runs shows that the phase transitions of all polymer solutions were fully reversible. 

As commonly observed, the cloud point temperatures of the 5 mg·mL^−1^ samples are always lower than those of the 1 mg·mL^−1^ samples [17]. Despite the relatively large difference in the balance of hydrophilic EtOx and hydrophobic MMA repeating units, the cloud point temperatures decrease only slightly with increasing percentage of the hydrophobic MMA monomer when graft copolymers with an identical side chain DP of 15 or 24 are compared (i.e., **P5** vs. **P6** or **P7** vs. **P8**). In addition, the polymers become completely insoluble very suddenly upon a slight increase of their hydrophobic character. This is evident from the comparison of the series **P4**–**P6** (Table 3). In addition, we noticed the same effect for graft polymers with the side chain DP of 5 (data not shown). Although the T_cp_ of aqueous solutions of these polymers can be increased above 35 °C by increasing the molar fraction of OEtOxMA [14], a straightforward cloud point tuning is no longer possible for OEtOxMA with a DG below 35%.

The cloud point temperatures of **P5**–**P8** are in the same range as for the homopolymer PEtOx and are, therefore, mostly dominated by the OEtOx side chains. As reference values, high molar mass PEtOx with cloud point temperatures in the range of 61 to 69 °C [25] or a block copolymer with nonyloxazoline (NonOx), i.e. EtOx_90_-*b*-NonOx_10_ with a T_cp_ of 69 °C [26] can be given. In particular, the latter example shows the influence of a hydrophobic end group, which results in a decreased T_cp_. Thus, it can be concluded that hydrophilic side chains of DP 15 and DP 24 both shield the hydrophobic backbone by making it inaccessible to the water molecules. In contrast, the shorter side chain length of DP 5 for **P1**–**P3** enables the penetration of the water molecules through the shell towards the hydrophobic core, which explains why the MMA content displays a strong influence on the T_cp_ in this case. This conclusion is further affirmed through comparison of the overall EtOx mol % of a graft copolymer, i.e., **P3** with 71 mol % EtOx and a T_cp_ of 35.4 °C vs. **P5** with only 65 mol % EtOx and a much higher T_cp_ of 59.9 °C (Table 3). Even though the total amount of hydrophilic EtOx units is similar, it is highly decisive in what way they are incorporated in the polymer structure, which hints towards the presence of differing structures in aqueous solution.

The influence of additional salts on the LCST behavior was investigated by measurement in a physiological buffer (Figures S5–S7). The T_cp_ values in PBS were roughly 4 °C lower than in water, which has also been found for other polymers types, such as cationic poly(ionic liquid)s [27]. This behavior is caused by the salting out effect of the ions present in the saline following the Hofmeister series. In particular, sodium hydrogen phosphate and sodium chloride are of major influence [28].

The LCST behavior of **P5** was further investigated by ^1^H NMR spectroscopy to verify this assumption. To enable a direct comparison of the data obtained by turbidimetry, the T_cp_ values for **P5** in D_2_O solution were determined as 59.8 °C and 57 °C at concentrations of 5 mg·mL^−1^ and 10 mg·mL^−1^, respectively. The variation compared to the T_cp_ in H_2_O is due to the energetic difference between hydrogen and “deuterium bonds” as hydrogen bonds represent the structural basis for LCST behavior in water [29,30].

**Figure 3:**
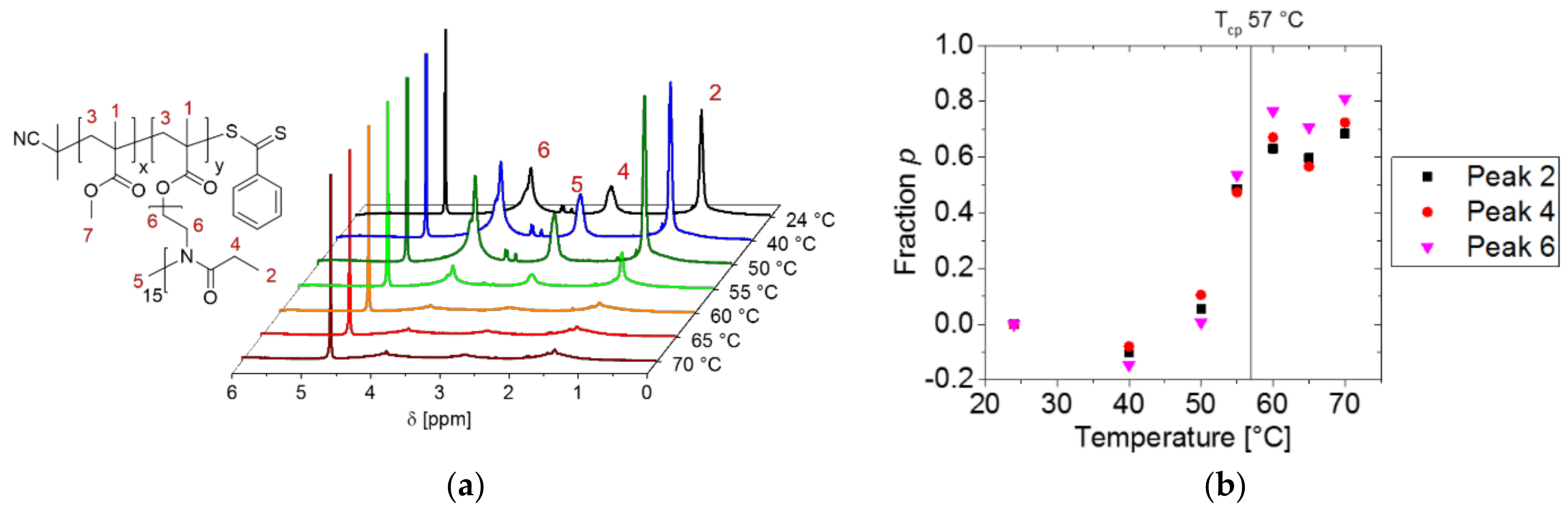
(**a**) ^1^H NMR spectra of **P5** in D_2_O at different temperatures (300 MHz, c = 10 mg·mL^−1^). (**b**) Temperature dependence of the calculated *p*-fraction of side chain units with significantly reduced mobility.

The ^1^H NMR spectra of **P5** in D_2_O at different temperatures are shown in Figure 3. The measurements were performed in a temperature range from 24 to 70 °C, i.e., above and below the T_cp_. The slight shift of the polymer peaks is caused by the use of the non-deuterated solvent signal as an internal standard [15]. Already the ^1^H NMR spectrum measured at room temperature revealed important information: in contrast to the ^1^H NMR spectrum measured in CDCl_3_ (Figure 2), peaks resulting from both the backbone as well as peaks assigned to the side chains are found; only peaks assigned to the OEtOx side chains of the graft copolymer can be seen in the ^1^H NMR spectrum measured in D_2_O. This hints towards the fact that the hydrophobic backbone of **P5** is immobilized and might suggest the presence of micelles comprising a core formed from the PMMA graft copolymer backbone.

The initial increase of the temperature (below the T_cp_) leads to sharper signals due to an increased mobility of the side chains. A further increase of the temperature above the T_cp_ of the solution leads to a pronounced broadening of the peaks and eventually their disappearance within the baseline, hinting towards the fact that the side chains collapsed upon phase transition. Even the methyl-signals of the OEtOx side chain end groups disappear, which indicates that the entire macromolecular structure is affected by the temperature change.

In order to allow a better evaluation of the structural changes during the phase transition, the integrals of the polymer signals were normalized according to the H_2_O signal and further utilized to calculate the values of the *p*-fraction of the EtOx units with significantly reduced mobility using Equation (1) [31].

(1) $$p=1-\left(\frac{I}{I_{0}}\right)$$

*I*_0_ corresponds to a signal integral at room temperature and *I* is the integral for the same signal at an elevated temperature below or above the T_cp_ (Figure 3b). At 55 °C, i.e., still below the T_cp_, the ^1^H NMR data revealed a significant decrease of all signals, regardless to their individual structural assignments. This indicates a simultaneous collapse of the entire side chain. A general statement can be made that at 70 °C, on average, 70% of the side chains are immobilized. Similar observations were made for the graft copolymer **P2** with a DG of 23% and a side chain DP of 5 in D_2_O (Figure S8).

**Figure 4:**
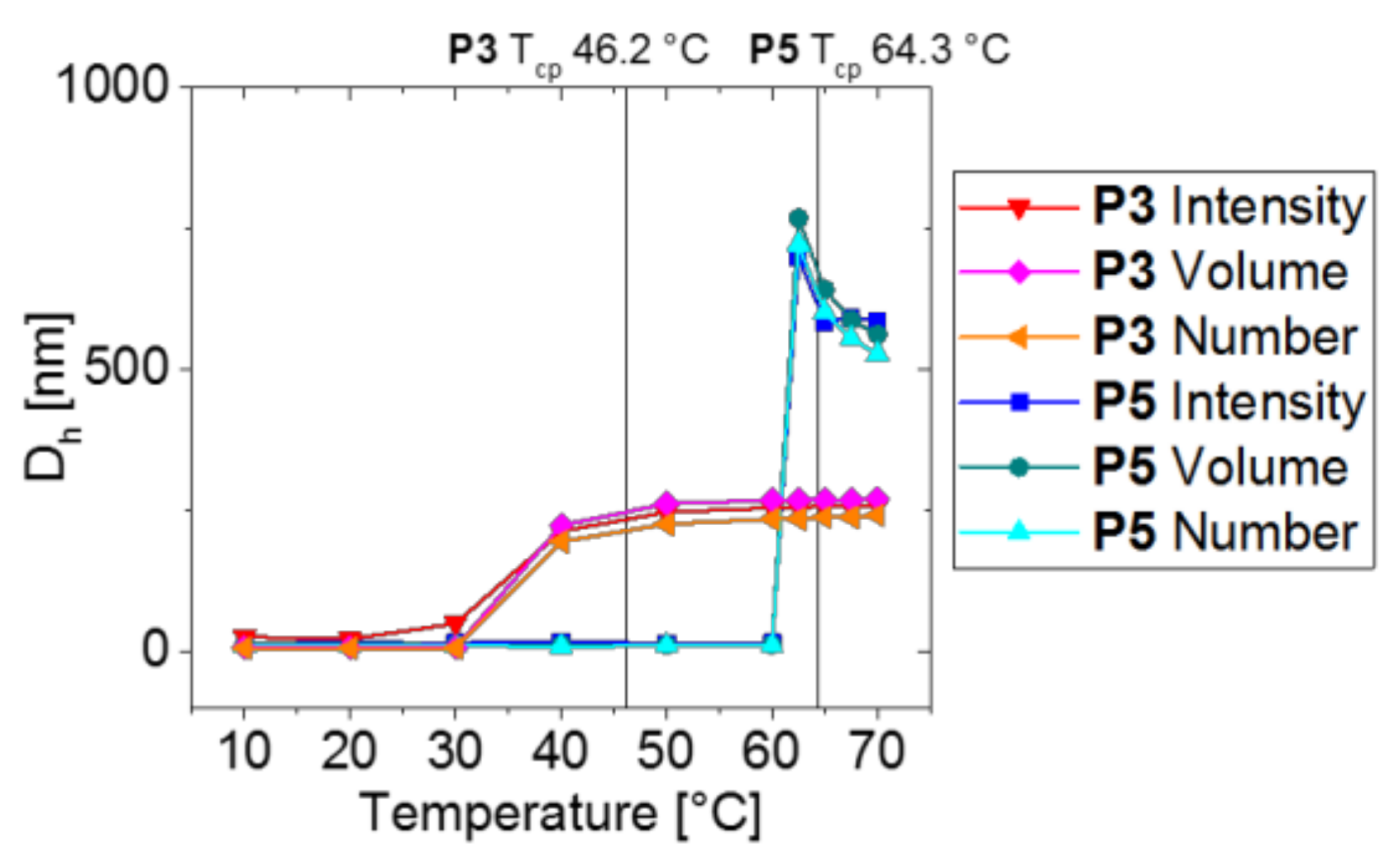
Temperature dependence of the hydrodynamic diameters (D_h_) of **P3** and **P5** as determined by dynamic light scattering (1 mg·mL^−1^ in deionized H_2_O). D_h_ is provided based on intensity, volume, and number distributions. The corresponding T_cp_ values of **P3** and **P5** as determined by turbidimetry measurements are also indicated. Lines are added to guide the eye.

To investigate the LCST behavior during the phase transition on a mesoscopic scale, aqueous solutions of **P3** (DG = 34, side chain DP = 5) and **P5** (DG = 11, side chain DP = 15) were investigated by DLS measurements at varying temperatures below and above T_cp_ at a polymer concentration of 1 mg·mL^−1^ in water (Figure 4). Both polymers show a heat-induced self-association, as can be seen from the increase in the apparent hydrodynamic diameter (D_h_) upon temperature increase for each polymer, regardless if the intensity, number, or volume based D_h_ distributions, are considered. However, the coil-to-globule transition of **P3** and **P5** differs significantly. For **P3**, an apparent D_h_ of 5 nm at 20 °C is observed. The D_h_ strongly increases to 250 nm at 40 °C and does not change significantly upon further temperature elevation. This behavior might be due to the formation of mesoglobules, i.e., colloidally stable aggregates with a spherical shape of more than one and less than all polymer chains. Mesoglobules are reported in literature above the LCST, for e.g., PNIPAM or poly(*N*,*N’*-diethyl acrylamide) [32], but also for poly(2-propyl-2-oxazoline) [33].

In contrast, the intensity based D_h_ distribution of **P3** at 20 °C is bimodal with peaks at 5 nm and 160 nm (Figure S9a). Upon phase transition, large aggregates were detected that shrink upon further temperature increase, as we previously observed for comb polymers comprising OEtOx side chains [34]. This behavior is known in literature, for e.g., poly(2-isopropyl-2-oxazoline)-*block*-poly(2-ethyl-2-oxazoline), and indicates the formation of concentrated-phase droplets in a diluted phase [35,36]. Presumably, the polymer concentration inside these droplets is increased at higher temperatures, which explains their shrinkage above the T_cp_. For both polymers **P3** and **P5**, the obtained DLS values for the onset of the phase transition are significantly lower than the cloud point temperatures determined via turbidimetry measurements, which shows that polymer agglomerates are formed even when they do not yet cause visible clouding of the solution.

### 3.4. Aggregation Behavior

**Figure 5:**
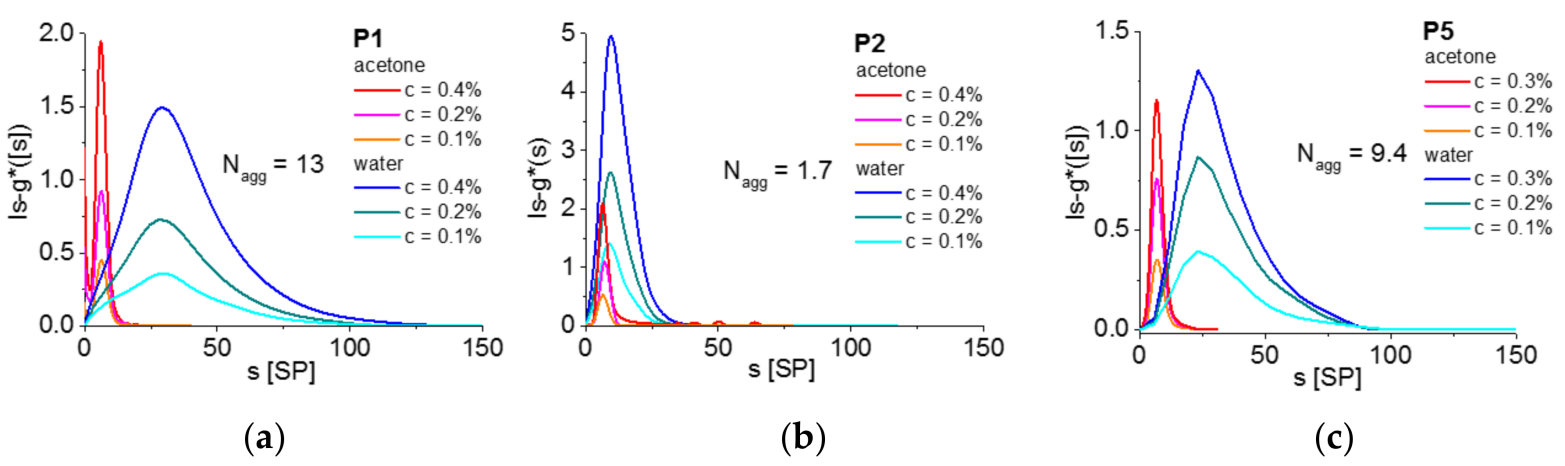
Differential distributions of intrinsic sedimentation coefficients [*s*] of the PMMA-*g*-OEtOx **P1** (**a**), **P2** (**b**)**,** and **P5** (**c**) at different concentrations obtained by sedimentation velocity experiments with analytical ultracentrifugation (AUC) in both acetone and deionized H_2_O as solvents, respectively.

As NMR and DLS investigations already hinted at a possible formation of micelles, in particular for **P5**, selected PMMA*-g*-OEtOx graft copolymers were further investigated by means of AUC to estimate the apparent aggregation number, N_agg_, in water. For this purpose, the apparent molar masses, M_s,f_, of the polymers and their aggregates were determined in acetone and water via sedimentation-diffusion analysis. The c(s) model in Sedfit [21] was used, providing numerical solutions to the Lamm equation, assuming the same weight average translational frictional ratio of the macromolecules and aggregates [37,38]. To assess the molar mass of the unimolecularly dissolved graft copolymers, the absolute molar masses were determined in acetone, representing a good solvent for the PMMA backbone as well as for the OEtOx side chains. Therefore, the absence of aggregates can be assumed. As evident from Table 4, the resulting molar masses obtained by sedimentation-diffusion analysis in acetone are in a good agreement with the theoretically expected values. Figure 5 depicts overlays of the apparent distributions of intrinsic sedimentation coefficients [s] from AUC of **P1**, **P2**, and **P5** without consideration of any diffusion effects (i.e., derived from the ls-g*(s) model resolving the apparent differential distribution of sedimentation coefficients, s, in each solvent). The overlay of the distributions of intrinsic sedimentation coefficients $\left[s\right]=s\eta_{0}/\left(1-\upsilon \varrho_{0}\right)$ with s being the actual sedimentation coefficients, $\eta_{0}$ being the dynamic viscosity of the solvent (acetone or water), υ being the partial specific volume of the polymer or aggregate, and $\varrho_{0}$ being the solvent density (acetone or water), was used to account for solvent effects. Therefore, it directly allows comparison of sedimentation behavior void of the influence of the type of solvent.

It is apparent for all three polymers that the corresponding distributions of intrinsic sedimentation coefficients, [s], on first sight are broader and shifted to higher values in water when compared to those of acetone. This clearly indicates an aggregation in water already below T_cp_, since centrifugation experiments were performed at 20 °C. The DG of the graft copolymers **P1** and **P2** featuring a side chain DP of 5 differs only by 6 mol %. However, the apparent aggregation number, N_agg_, in water is observed being significantly influenced by this small change. The more hydrophobic **P1** formed aggregates of 13 macromolecules on average, whereas the only slightly more hydrophilic **P2** appeared approximately unimolecular to dimeric (N_agg_ = 1.7). Apparently, a DG of 23% with these short side chains is almost sufficient to shield the hydrophobic backbone from surrounding water molecules. This variation could probably explain why the T_cp_ of the PMMA-*g*-EtOx_5_ solutions is cumbersome to fine-tune in this composition range. Although the graft copolymer **P5** (with a side chain DP of 15) features an even higher fraction of hydrophilic EtOx repeating units than **P2**, it is far from being present as unimolecular in aqueous solution (N_agg_ = 9.4). Apparently, due to the increased conformational freedom of a graft copolymer with lower DG but higher side chain DP, several macromolecules assemble into micelles featuring a PMMA core and shell formed by the OEtOx side chains. Despite being relatively insensitive to concentration effects within the investigated range, these assemblies are expected to be dynamic because of their non-covalent nature, which would explain the broad distribution of sedimentation coefficients clearly reaching into the unimolecularly dissolved region. Such results are in agreement with the reported research from Košovan et al. on the molecular dynamics simulations and scaling theory on the case study of amphiphilic graft copolymers in selective solvents [19].

### 3.5. Dye Encapsulation

The fact that some PMMA-*g*-OEtOx formed assemblies comprising a hydrophobic core that is shielded by OEtOx could potentially make them carrier systems for hydrophobic drugs. This was tested utilizing the hydrophobic dye Disperse Orange 3 (DO3) as a model guest molecule. The encapsulation was performed five times for each polymer with varying molar ratios of polymer:dye from 1:1 to 1:5. Simultaneously, five control samples without polymer were prepared, to avoid falsification of the results through dissolution of small amounts of the hydrophobic dye in aqueous solvents. Figure S10 shows the calibration curve for DO3 in acetone. The resulting data are provided in Table 3 as molar ratios, i.e., *n* molecules of dye were solubilized per macromolecule. The highest loading of 1.9 was achieved for **P3** hinting towards the fact that the less defined aggregates of **P3** solubilize the dye more efficiently than more well-defined aggregates formed from graft copolymers comprising longer side chains. Although **P6** and **P8** differ in side chain DP and DG, the molar fraction of EtOx is almost identical. Both PMMA-*g*-OEtOx solubilize the same amount of DO3. For graft copolymers with the same side chain DP, the DG clearly impacts the dye uptake. The less hydrophobic graft copolymer **P6** (DG = 24) can solubilize less dye than the more hydrophobic **P5** (DG = 11). One might assume that a higher molar fraction of MMA would lead to a larger hydrophobic core of a micelle formed in water and, therefore, promote uptake of a larger amount of a hydrophobic dye.

## 4. Conclusions

A series of PMMA-*g*-OEtOx graft copolymers containing a hydrophobic poly(methyl methacrylate) (PMMA) backbone and hydrophilic oligo(2-ethyl-2-oxazoline) (OEtOx) side chains were synthesized via reversible addition-fragmentation chain transfer (RAFT) copolymerization of MMA and OEtOx-methacrylate macromonomers of varying DPs. The graft copolymer architecture significantly influenced the aggregation behavior, the conformation in aqueous solution, as well as the coil to globule transition, as verified by means of turbidimetry, dynamic light scattering, nuclear magnetic resonance spectroscopy, and sedimentation analysis via analytical ultracentrifugation. The aggregation behavior of graft copolymers with a side chain DP of 5 was significantly affected by small variations of the copolymer composition, i.e., the DG, occasionally forming mesoglobules above the cloud point temperature (T_cp_) which was around human body temperature. On the other hand, PMMA-*g*-OEtOx with elongated side chains assembled into well-defined structures below the T_cp_ and were capable of solubilizing the hydrophobic model dye DO3. Current research in our laboratories is directed towards the application of PMMA-*g*-OEtOx assemblies as drug carriers, benefiting from the biocompatible PEtOx-based shell exhibiting a stealth effect.

## Figures and Tables

**Table 1:** Characterization results for the OEtOxMA macromonomers **MM1**–**MM3**.

MM ^1^	M/I	DP ^2^	M_n_^2^ (g·mol^−1^)	M_n_^3^ (g·mol^−1^)	Ð ^3^
**MM1**	5	5	600	500	1.12
**MM2**	15	15	1600	1300	1.11
**MM3**	20	24	2500	1700	1.15

^1^ Quantitative monomer conversion. ^2 1^H NMR (CDCl_3_, 300 MHz). ^3^ Size exclusion chromatography (SEC) (CHCl_3_, PMMA calibration, refractive index (RI) detection). M/I = [monomer] to [initiator] ratio; DP = degree of polymerization; M_n_ = molar mass; Ð = dispersity.

**Table 2:** Characterization results for the graft copolymers PMMA-*g*-OEtOx.

P	MM	MM/MMA/CTA ^1^	mol % MM/MMA (feed)	Conv. (%) ^2^	M_n_ (g·mol^−1^) ^3^	Ð ^3^	mol % MM/MMA (NMR) ^4^	DP MM/MMA ^5^
**P1**	**MM1**	30:80:1	27/73	63	12,900	1.32	17/83	12/57
**P2**	**MM1**	30:60:1	33/67	90	13,400	1.19	23/77	19/62
**P3**	**MM1**	30:60:1	33/67	88	14,000	1.09	34/66	27/52
**P4**	**MM2**	13:77:1	14/86	64	10,900	1.17	9/91	5/53
**P5**	**MM2**	18:72:1	20/80	88	14,800	1.13	11/89	9/70
**P6**	**MM2**	30:60:1	33/67	56	13,000	1.13	24/76	12/38
**P7**	**MM3**	18:72:1	20/80	86	13,600	1.11	9/91	7/71
**P8**	**MM3**	30:60:1	33/67	81	13,000	1.13	18/82	13/60

^1^ Feed ratio. ^2^ Calculated from ^1^H NMR spectra (CDCl_3_, 300 MHz) using *N,N*-dimethylformamide (DMF) as internal standard. ^3^ SEC (CHCl_3_, PMMA calibration, RI detection). ^4^ Calculated from the ^1^H NMR spectra (CDCl_3_, 300 MHz) of the purified polymers, the degree of grafting corresponds to the mol % of **MM**. ^5^ Calculated from the used [monomer]/[chain transfer agent] (M/CTA) ratio, the overall monomer conversion and the composition of the purified polymers.

**Table 3:** Cloud point temperatures of PMMA-*g*-OEtOx **P1**–**P8** in aqueous solution.

Polymer		DP Side Chain	DG ^1^	mol % MM/MMA ^1^	mol % EtOx/MMA	T_cp_ (°C) ^2^ in H_2_O		DO3/P Uptake ^3^ (mol_(DO3)_·mol_(P)_^−1^)
						c (mg·mL^−1^)		
						5	1	
**P1**	polyMMA_57_-*g*_17%_-polyEtOx_5_	5	17	17/83	51/49	37	- ^4^	n.d.
**P2**	polyMMA_62_-*g*_23%_-polyEtOx_5_	5	23	23/77	60/40	40.5	48.8	n.d.
**P3**	polyMMA_52_-*g*_34%_-polyEtOx_5_	5	34	34/66	71/29	35.4	45.2	1.9
**P4**	polyMMA_53_-*g*_9%_-polyEtOx_15_	15	9	9/91	56/44	not water soluble		n.d.
**P5**	polyMMA_70_-*g*_11%_-polyEtOx_15_	15	11	11/89	65/35	59.9	63.05	1.7
**P6**	polyMMA_38_-*g*_24%_-polyEtOx_15_	15	24	24/76	83/17	62.9	66.6	1.2
**P7**	polyMMA_71_-*g*_9%_-polyEtOx_24_	24	9	9/91	68/32	61.9	66.8	n.d.
**P8**	polyMMA_60_-*g*_18%_-polyEtOx_24_	24	18	18/82	84/16	67.1	70.6	1.2

^1^ Degree of grafting (DG) corresponds to the mol % of **MM**. ^2^ Heating rate 1 K·min^−1^, average value of two heating ramps at 50% transmittance. ^3^ Dye encapsulation results for graft copolymers with the dye Disperse Orange 3 (DO3) in deionized H_2_O given in mol dye per mol polymer for the highest determined molar ratio of 1:5 at a c(P) = 0.6 mg·mL^−1^. ^4^ Only slight decrease in transmittance. T_cp_ = cloud point temperature.

**Table 4:** Summary of the characterization results of AUC for **P1**, **P2,** and **P5**.

Polymer	Side Chain DP	mol % MM/MMA ^1^	mol % EtOx/MMA	M_n, theor._^2^ (g·mol^−1^)	M_sf(acetone)_ (g·mol^−1^)	M_sf(water)_ (g·mol^−1^)	N_agg_^3^
**P1**	5	17/83	51/49	13,100	15,200	196,000	13
**P2**	5	23/77	60/40	17,800	18,800	31,800	1.7
**P5**	15	11/89	65/35	21,600	24,700	231,800	9.4

^1^ mol % **MM** corresponds to the degree of grafting. ^2^ Calculated with the determined copolymer composition and monomer conversion. ^3^ Aggregation number in water obtained by dividing M_s,f(acetone)_ by M_s,f(water)_. M_s,f_ = apparent molar masses.

## Supplementary Materials

The following are available online at http://www.mdpi.com/1996-1944/11/4/528/s1, Extended experimental section describing the polymerization conditions for the individual macromonomers and polymers, Figure S1: ^1^H NMR spectrum with enlarged intensities of **P5**, Figure S2: Turbidimetry curves for **P1**–**P3**, Figure S3: Turbidimetry curves for **P5**–**P6**, Figure S4: Turbidimetry curves for **P7**–**P8**, Figure S5: Turbidimetry curves in PBS for **P2**–**P3**, Figure S6: Turbidimetry curves in PBS for **P5**–**P6**, Figure S7: Turbidimetry curves in PBS for **P7**–**P8**, Figure S8: ^1^H NMR spectra of **P2** in D_2_O at different temperatures, Figure S9: Intensity-weighted DLS size distributions detected at varying temperatures below and above the T_cp_ in aqueous solutions of **P3** and **P5**, Figure S10: Calibration data for the quantification of Disperse Orange 3 (DO3) by UV Vis absorption spectroscopy in acetone at 438 nm.
