# Supplementary material for: PMMA-g-OEtOx Graft Copolymers: Influence of Grafting Degree and Side Chain Length on the Conformation in Aqueous Solution
Source: Materials (Basel). 2018 Mar 30;11(4):528. doi: 10.3390/ma11040528 (PMC5951374; doi:10.3390/ma11040528)
Supplement: Supplementary file 1 [file materials-11-00528-s001.pdf]

materials

Supporting Information

PMMA-*g*-OEtOx Graft Copolymers: Influence of Grafting Degree and Side Chain Length on the Conformation in Aqueous Solution

Irina Muljajew[1,2], Christine Weber[1,2*], Ivo Nischang[1,2] and Ulrich S. Schubert[1,2,*]

1 Laboratory of Organic and Macromolecular Chemistry (IOMC), Friedrich Schiller University Jena, Humboldtstr. 10, 07743 Jena, Germany
2 Jena Center for Soft Matter (JCSM), Friedrich Schiller University Jena, Philosophenweg 7, 07743 Jena, Germany
* Correspondence: ulrich.schubert@uni-jena.de (U.S.S.), Tel.: +49-3641-948200; christine.weber@uni-jena.de (C.W.)

Full experimental section describing the macromonomer synthesis:

General macromonomer synthesis procedure. The macromonomers (**MM**) were synthesized according to a modified procedure previously published. MeTos, EtOx, and acetonitrile were transferred into a preheated vial under inert conditions. The concentration of EtOx was 4 mol L^{-1}, and the total reaction solution volume was 15 mL. The polymerization was performed in the microwave at 140 °C to reach a ln([M]$_0$/[M]$_t$) of 4 according to the k_p value of 0.255 L mol^{-1} s^{-1}. Subsequently, a 1.5-fold excess of methacrylic acid (MAA) and a 2-fold excess of triethyl amine (NEt$_3$) were added *via* syringe through the septum of the vial (excess by reference to the initiator). The reaction solution was kept at 50 °C overnight to allow for end functionalization. The reaction mixture was dissolved in chloroform (100 mL), washed with saturated aqueous sodium bicarbonate solution (2 × 100 mL) and brine (2 × 100 mL), dried over sodium sulfate, and concentrated under reduced pressure at 30 °C. The honey-like pale yellow product was stored at –20 °C.

MM1: Aiming at a [MeTos]:[EtOx] ratio of 1:5, **MM1** was obtained according to the general procedure using 2.23 g (12 mmol) MeTos, 5.95 g (60 mmol) EtOx, 8.94 mL acetonitrile, 1.55 g (18 mmol) MAA, 2.42 g (24 mmol) NEt$_3$ applying a polymerization time of 20 s. ^{1}H NMR (CDCl$_3$, 300 MHz): δ/ppm = 1.10 (15H, H-1), 1.89 (3H, H-2), 2.17-2.48 (10H, H-3), 3.00 (3H, H-4), 3.28-3.72 (18H, H-5), 4.24 (2H, H-6), 5.56 (1H, H-7), 6.05 (1H, H-8). SEC (CHCl$_3$/*i*PrOH/TEA, RI detection, PMMA calibration): M_n = 500 g mol^{-1}, Đ = 1.12.

MM2: Corresponding to a [MeTos]:[EtOx] ratio of 1:15, **MM2** was obtained according to the general procedure using 0.74 g (4 mmol) MeTos, 5.95 g (60 mmol) EtOx, 8.97 mL acetonitrile, 0.52 g (6 mmol) MAA, 0.81 g (8 mmol) NEt$_3$ employing a polymerization time of 60 s. ^{1}H NMR (CDCl$_3$, 300 MHz): δ/ppm = 1.11 (47H, H-1), 1.91 (3H, H-2), 2.11-2.60 (31H, H-3), 3.01 (3H, H-4), 3.11-3.82 (59H, H-5), 4.26 (2H, H-6), 5.58 (1H, H-7), 6.06 (1H, H-8). SEC (CHCl$_3$/*i*PrOH/TEA, RI detection, PMMA calibration): M_n = 1300 g mol^{-1}, Đ = 1.11.

MM3: Aiming at a [MeTos]:[EtOx] ratio of 1:20, **MM3** was synthesized according to the general procedure using 0.56 g (3 mmol) MeTos, 5.95 g (60 mmol) EtOx, 8.94 mL acetonitrile, 0.39 g (4.5 mmol) MAA, 0.61 g (6 mmol) NEt$_3$ setting the polymerization time to 90 s. ^{1}H NMR (CDCl$_3$, 300 MHz): δ/ppm = 1.10 (72H, H-1), 1.91 (3H, H-2), 2.18-2.53 (47H, H-3), 3.00 (3H, H-4), 3.10-3.77 (93H, H-5), 4.25 (2H, H-6), 5.58 (1H, H-7), 6.06 (1H, H-8). SEC (CHCl$_3$/*i*PrOH/TEA, RI detection, PMMA calibration): M_n = 1700 g mol^{-1}, Đ = 1.18.

Full experimental section describing the RAFT polymerization:

General synthesis procedure for RAFT copolymerization. The respective macromonomers **MM1–MM3** and MMA were dissolved in ethanol in the desired ratio at an overall monomer concentration [M] of 1 mol L^{-1}. Subsequently, the initiator AIBN and the chain transfer agent 2-cyano-2-propyl benzodithioate (CPDB) were added from adequate stock solutions to achieve a [M]:[CPDB]:[AIBN] ratio of 90:1:0.25, unless noted otherwise. One equivalent of *N,N*-dimethylformamide (DMF) with respect to **MM** was added as internal standard. The reaction solution was gently degassed by argon bubbling through the septum of the closed vial for 30 min. A t_0 sample was taken to determine the monomer conversion by means of ^{1}H NMR spectroscopy. The vial was heated to 70 °C in an oil bath overnight and another sample was taken. The reaction solution was concentrated under reduced pressure and subsequently purified by preparative size exclusion chromatography (BioBeads SX-1 in THF). The desired fractions were concentrated under reduced pressure, the product was precipitated into cold diethyl ether and dried under reduced pressure at 40 °C. The purified polymers were characterized by means of ^{1}H NMR spectroscopy and SEC (compare Table 2).

^{1}H NMR ($CDCl_3$, 300 MHz): *δ*/ppm = 1.51-0.63 (H-1, H-2), 2.12-1.58 (H-3), 2.14-2.58 (H-4), 2.86-3.16 (H-5), 3.16-3.77 (H-6,H-7), 3.88-4.20 (H-6).

P1: According to a [**MM1**]:[MMA] ratio of 1:2.7, 1.05 g (1.8 mmol) **MM1**, 0.45 g (4.4 mmol) MMA, 2.3 mg (14 µmol) AIBN, 12.3 mg (60 µmol) CPDB and 11.76 mL ethanol were used.

P2: According to a [**MM1**]:[MMA] ratio of 1:2, 6.00 g (10 mmol) **MM1**, 2.00 g (20 mmol) MMA, 13.7 mg (83 µmol) AIBN, 73.8 mg (33 µmol) CPDB and 15.97 mL ethanol were used.

P3: According to a [**MM1**]:[MMA] ratio of 1:2, 1.98 g (3.3 mmol) **MM1**, 0.67 g (6.7 mmol) MMA, 4.6 mg (28 µmol) AIBN, 24.6 mg (111 µmol) CPDB and 9.0 mL ethanol were used.

P4: According to a [**MM2**]:[MMA] ratio of 1:6, 1.0 g (0.6 mmol) **MM2**, 0.4 g (3.8 mmol) MMA, 2.0 mg (12 µmol) AIBN, 10.8 mg (49 µmol) CPDB and 4.0 mL ethanol were used.

P5: According to a [**MM3**]:[MMA] ratio of 1:4, 1.0 g (0.6 mmol) **MM3**, 252.4 mg (2.5 mmol) MMA, 1.4 mg (8.8 µmol) AIBN, 7.7 mg (35 µmol) CPDB and 2.8 mL ethanol were used.

P6: According to a [**MM2**]:[MMA] ratio of 1:2, 1.0 g (0.6 mmol) **MM2**, 126 mg (1.3 mmol) MMA, 0.9 mg (5.3 µmol) AIBN, 4.6 mg (21 µmol) CPDB and 1.7 mL ethanol were used.

P7: According to a [**MM3**]:[MMA] ratio of 1:4, 1.0 g (0.4 mmol) **MM3**, 155.5 mg (1.6 mmol) MMA, 0.9 mg (5.4 µmol) AIBN, 4.8 mg (21.6 µmol) CPDB and 1.7 mL ethanol were used.

P8: According to a [**MM3**]:[MMA] ratio of 1:2, 1.0 g (0.4 mmol) **MM3**, 77.7 mg (0.8 mmol) MMA, 0.5 mg (3.2 µmol) AIBN, 2.9 mg (13 µmol) CPDB and 1.1 mL ethanol were used.

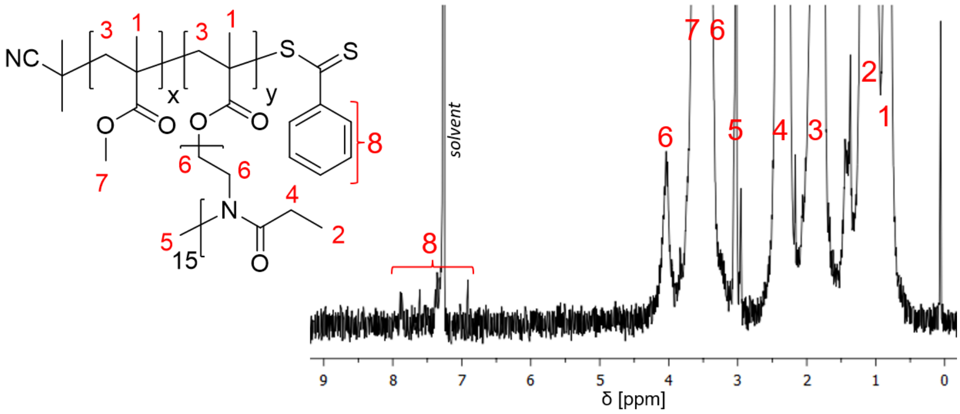

Figure S1. ^{1}H NMR spectrum ($CDCl_3$, 300 MHz) of the PMMA-*g*-OEtOx **P5** including the assignment of the benzodithioate end group signals.

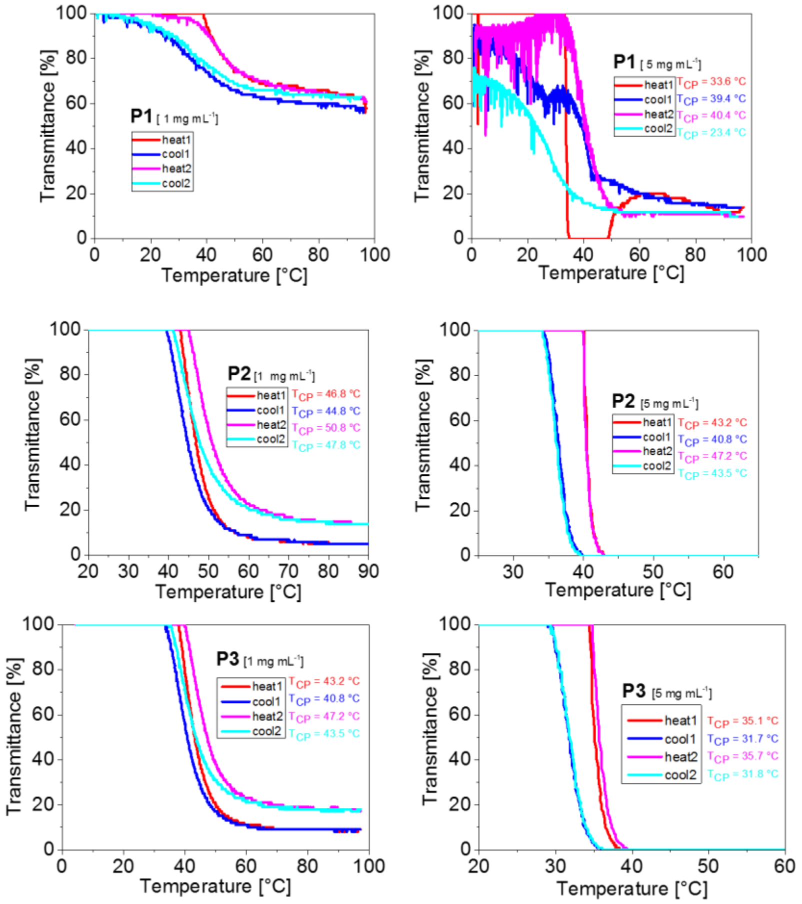

Figure S2. Turbidimetry curves for **P1**–**P3** (1 and 5 mg·mL^{-1} in H_2O, heating rate 1 K·min^{-1}).

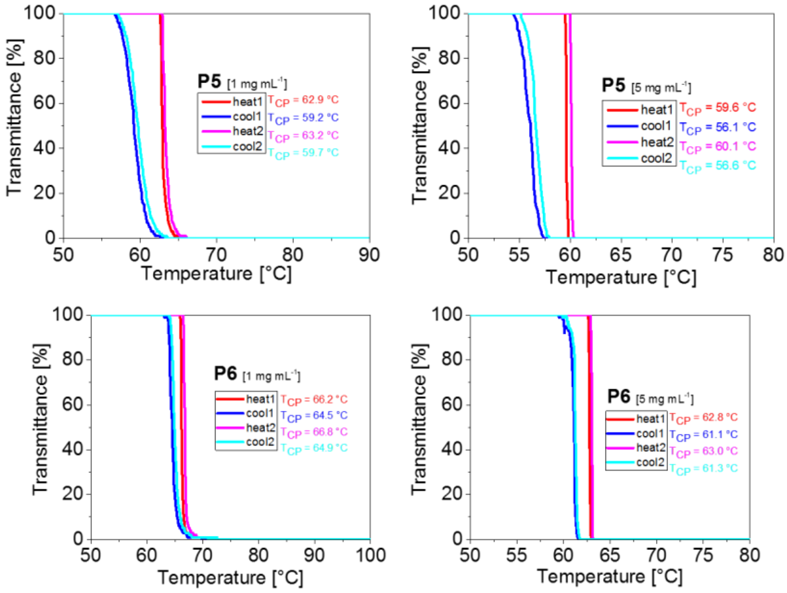

Figure S3. Turbidimetry curves for **P5** and **P6** (1 and 5 mg·mL^{-1} in H_2O, heating rate 1 K·min^{-1}).

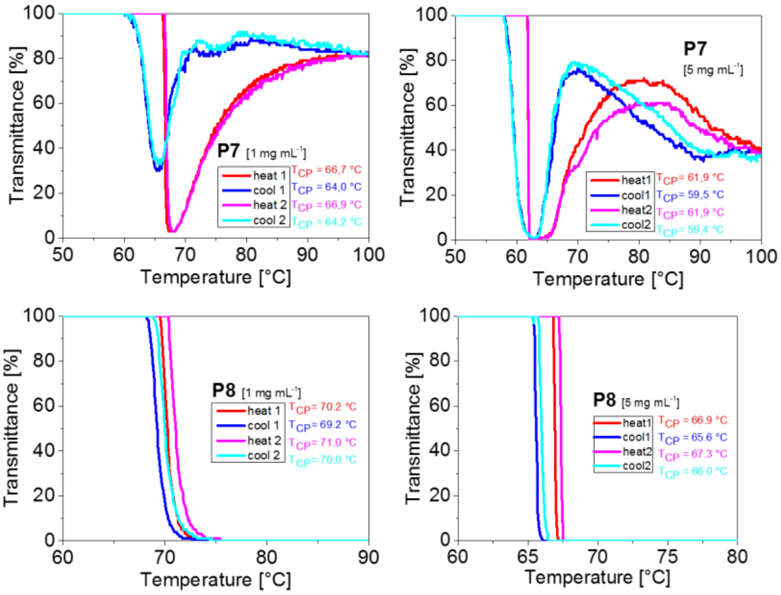

Figure S4. Turbidimetry curves for **P7** and **P8** (1 and 5 mg·mL^{-1} in H_2O, heating rate 1 K·min^{-1}).

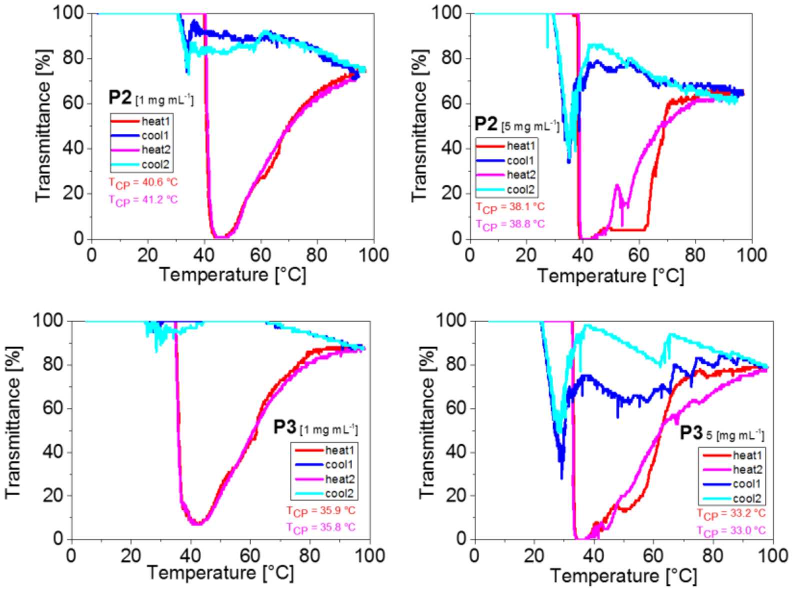

Figure S5. Turbidimetry curves for **P2** and **P3** (1 and 5 mg·mL^{-1} in PBS, heating rate 1 K·min^{-1}).

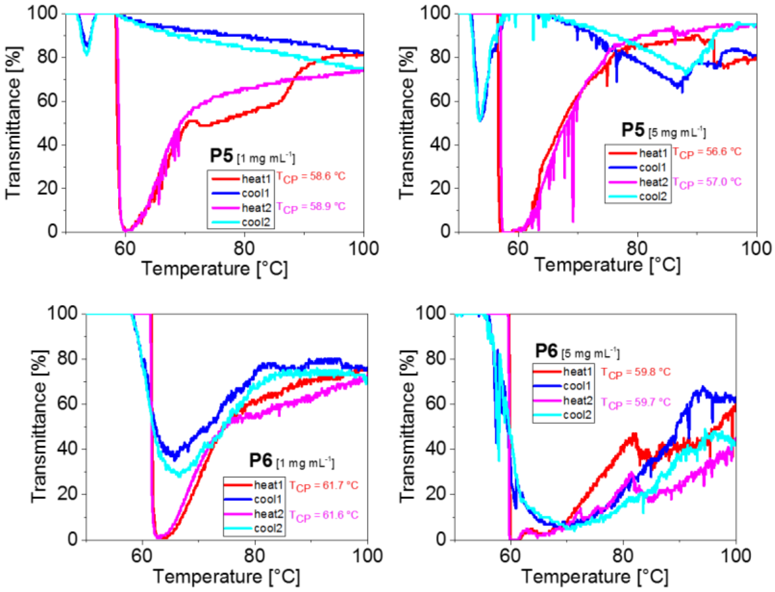

Figure S6. Turbidimetry curves for **P5** and **P6** (1 and 5 mg·mL^{-1} in PBS, heating rate 1 K·min^{-1}).

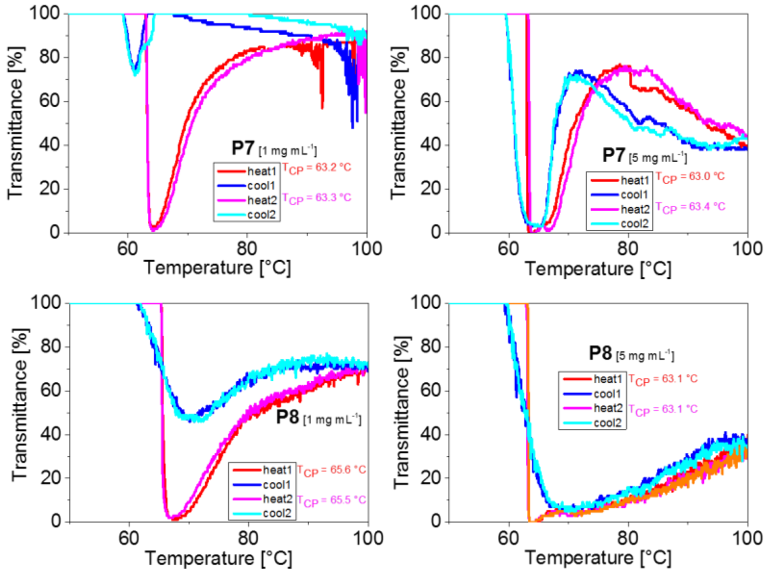

Figure S7. Turbidimetry curves for **P7** and **P8** (1 and 5 mg·mL^{-1} in PBS, heating rate 1 K·min^{-1}).

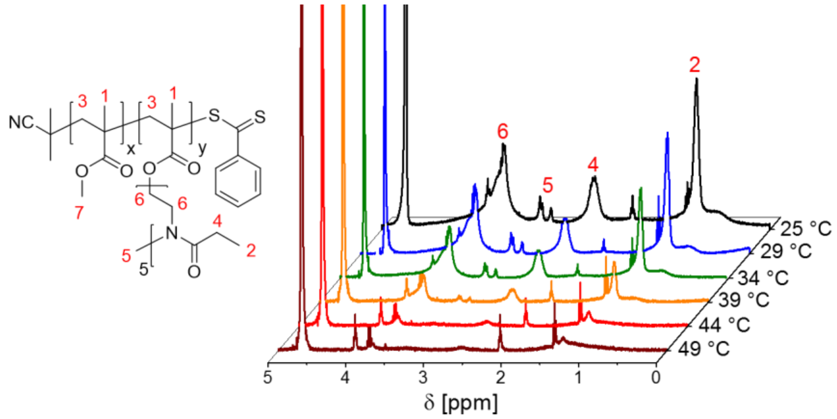

Figure S8. ^{1}H NMR spectra of **P2** in D_2O at different temperatures (400 MHz, c = 5 mg·mL^{-1}).

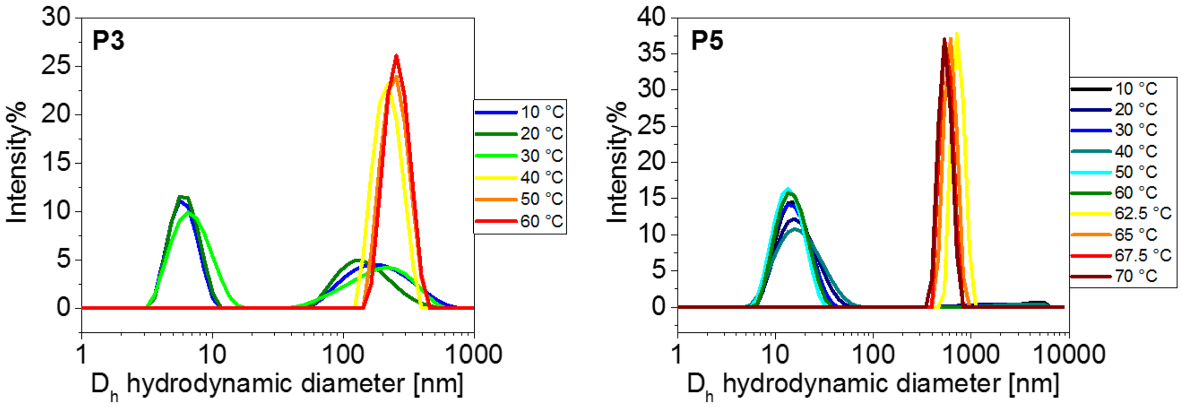

(**a**) (**b**)

Figure S9. Intensity-weighted dynamic light scattering (DLS) size distributions detected at varying temperatures below and above the T_{cp} in aqueous solutions of (**a**) **P3** and (**b**) **P5** (1 mg·mL^{-1} in deionized H_2O).

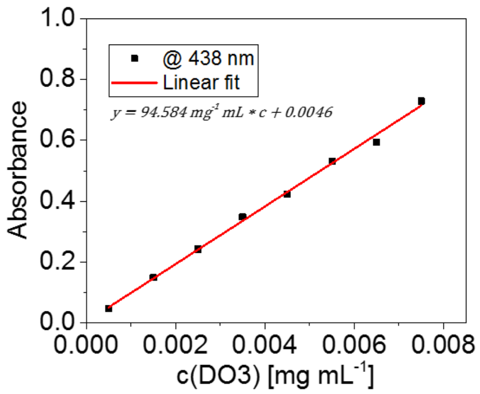

Figure S10. Calibration data for the quantification of Disperse Orange 3 (DO3) by UV Vis absorption spectroscopy in acetone at 438 nm. Graph and linear fit equation are shown.
